# A web-based support for pregnant women and new mothers with type 1 diabetes mellitus in Sweden (MODIAB-Web): study protocol for a randomized controlled trial

**DOI:** 10.1186/1745-6215-15-513

**Published:** 2014-12-29

**Authors:** Annsofie Adolfsson, Karolina Linden, Carina Sparud-Lundin, Per-Göran Larsson, Marie Berg

**Affiliations:** School of Health and Medical Sciences, Örebro University, SE 701 82 Örebro, Sweden; Institutes of Health and Care Science, Sahlgrenska Academy, Gothenburg University, Box 457, SE 405 30 Gothenburg, Sweden; Centre for Person-Centred Care (GPCC), University of Gothenburg, Box 457, SE 405 30 Gothenburg, Sweden; Department of Obstetrics and Gynecology, Skaraborg Hospital Skövde, SE 541 85 Skövde, Sweden

**Keywords:** Diabetes mellitus, type 1, Pregnancy, Support, Social media, Access information, Parenting, Motherhood, Randomized controlled trial

## Abstract

**Background:**

Women with type 1 diabetes face particular demands in their lives in relation to childbearing. During pregnancy, in order to optimize the probability of giving birth to a healthy child, their blood glucose levels need to be as normal as possible. After childbirth, they experience a ‘double stress’: in addition to the ordinary challenges they face as new mothers, they also need to focus on getting their blood glucose levels normal. To improve self-management of diabetes and overall well-being in women with type 1 diabetes, a person-centered web-based support was designed to be tested in a randomized controlled trial (RCT) to be used during pregnancy and early motherhood. This protocol outlines the design of this RCT, which will evaluate the effectiveness of the specially designed web-based support for mothers with type 1 diabetes in Sweden.

**Methods/Design:**

The study is designed as an RCT. The web support consists of three parts: 1) evidence-based information, 2) a self-care diary, and 3) communication with peers. The primary outcome is general well-being evaluated with the Well-Being Questionnaire short version (W-BQ12) and diabetes management evaluated with the Diabetes Empowerment Scale, short version (SWE-DES). Women attending six hospital-based antenatal care centers in Sweden are invited to participate. The inclusion period is November 2011 to late 2014. The allocation of participants to web support (intervention group) and to usual care (control group) is equal (1:1). In total, 68 participants in each group will be needed to reach a statistical power of 80% with significance level 0.05.

**Discussion:**

The web support is expected to strengthen the women’s personal capacity and autonomy during pregnancy, breastfeeding, and early motherhood, leading to optimal well-being and diabetes management.

**Trial registration:**

ClinicalTrials.gov: NCT01565824 (registration date March 27th 2012).

## Background

Maternal type 1 diabetes complicates pregnancy, childbirth and the time just after childbirth. Consequences for the fetus/newborn include congenital malformations, miscarriages, growth anomalies and stillbirth, fetal macrosomia, operational deliveries, neonatal complications and also perinatal mortality. Maternal concerns include increased likelihood of worsened diabetes-related vascular complications, pre-eclampsia and eclampsia, and of caesarian section and vacuum extraction/forceps vaginal delivery [1, 2]. The degree of complications is mainly related to maternal glycemia, where abnormal blood glucose levels increase adverse outcomes. Optimized maternal normoglycemia from pre-conception through pregnancy is the most important tool available to minimize complications and increase the likelihood that a woman will give birth to a healthy child [1, 3]. During pregnancy, all women have a physiological need for more insulin than usual (up to two or three times more). For women with diabetes treated with insulin, this means that, in order to optimize the situation for the coming offspring, they need to parry the successively increased insulin need through intensive blood glucose controls and continuous insulin dose increases combined with a structured diet and activity plan [2–4]. Directly after childbirth the insulin need decreases, often to less than the pre-gestational levels [5].

Diabetes is unique in that much of the day-to-day responsibility for care rests with the individuals who have the disease. Our earlier research in Sweden has highlighted the vulnerable situation for women with type 1 diabetes in relation to childbearing. During pregnancy, they struggle day and night to achieve normal blood glucose levels in an attempt to ensure that the fetus/child will be born healthy; they typically experience frequent hypoglycemic episodes and feelings of stress, worry and insecurity [6–8]. It has been found that this demanding situation is worsened if care providers lack competence and also if the mothers feel left aside by healthcare workers who give more attention to the unborn child than to the women themselves. After childbirth, the intensive health care provided during pregnancy and childbirth is interrupted, placing the new mothers in a vulnerable situation as they experience unstable glycemia and more hypoglycemic episodes in relation to breastfeeding [8]. The mothers also described that they down-prioritized their own needs in favor of the child’s [9]. Compared to mothers without diabetes, they were more affected by daily life disruptions [10] and reported lower levels of well-being [11].

A structured literature review of 16 identified studies performed in Sweden, USA, Australia, Greece, Israel, Italy and the UK [12] has indicated similar results. Women with type 1 diabetes who were in transition to motherhood experienced a variety of psychosocial issues: increased levels of anxiety, diabetes-related distress, guilt, a sense of disconnectedness from health professionals, and a focus on the medicalization of pregnancy. A trusting relationship with health professionals, sharing experiences with other women with diabetes, active social support, and shared decision making and responsibilities for diabetes management helped them achieve a positive transition to motherhood. The authors concluded that health professionals can promote a positive transition to motherhood by proactively supporting women with type 1 diabetes in informed decision making, by facilitating communication with the health-care team and by coordinating care [12].

Thus existing research shows that the period of pregnancy, childbirth and early motherhood is characterized by an increased risk of complications in both the woman with type 1 diabetes and the fetus/child, and that the woman experiences a complex everyday situation and a lower sense of well-being. There is an obvious need for extended person-support - from professionals, family and friends and also peers, that is, mothers with similar experiences of diabetes and childbearing.

Our own research shows that the use of Internet communication is needed and expected by childbearing women with type 1 diabetes [13]. Therefore, we suggest that a person-centered web-based support - one that is available 24/7 and enables users to document their experiences and share in decision-making related to their care - as a complement to usual health-care arrangements may strengthen self-management capabilities and thus well-being in women with type 1 diabetes during pregnancy, childbirth and early motherhood. To our knowledge, such a person-centered web-based support has not yet been evaluated.

### Objective

This protocol outlines the design of a specially created web-based support system for women with type 1 diabetes during pregnancy and in early motherhood, tested in a randomized controlled trial (RCT).

## Methods/Design

### Study design

**Figure 1:**
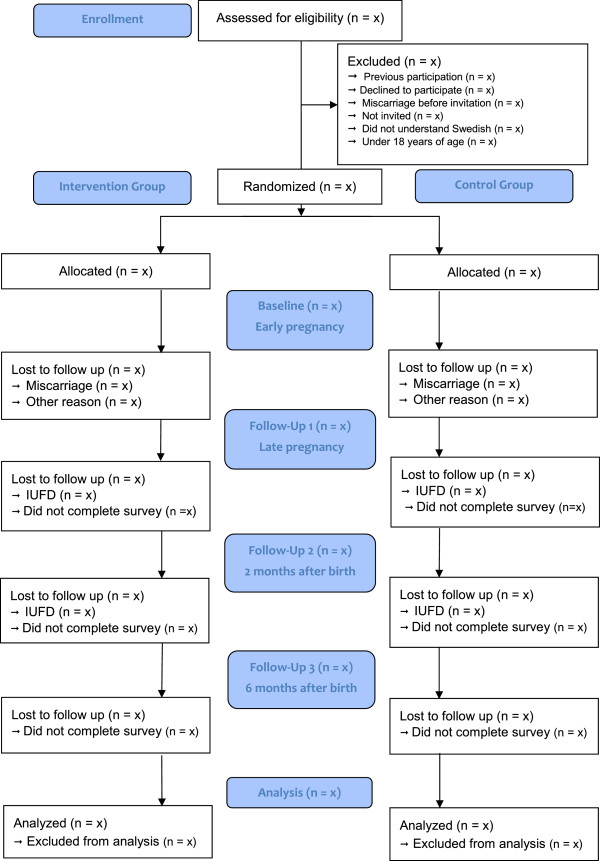
**Flow diagram MODIAB-Web evaluating a web-based support for pregnant women and new mothers with type 1 diabetes mellitus in Sweden: study protocol for a randomized controlled trial.**

The study is labeled MODIAB-Web and is designed as an RCT to evaluate the effect of a web-based support system on well-being and diabetes management in the study group. An equal number of study participants, pregnant women with type 1 diabetes, will be included in the intervention group and control group (1:1). Web-based support will be offered to the intervention group as a complement to the usual diabetes and pregnancy care. Women allocated to the control group will receive the usual care. The study design is in accordance with the CONSORT recommendation for reporting pragmatic randomized controlled trials [14]. The flow of the RCT is illustrated in Figure 1.

### Sample size estimation

Sample size estimation in this RCT is based on the two primary outcome variables: 1) general well-being measured by the Well-Being Questionnaire (W-BQ12) and 2) diabetes management measured by the Swedish Diabetes Empowerment Scale (SWE-DES). In order to detect a clinically relevant difference of 1.25 in well-being between the intervention group and the control group, 68 subjects will be needed in each group to reach a statistical power of 80%, assuming a SD of 0.40 in each group, with a significance level 0.05. Similarly, to detect a difference of 0.2 in diabetes management, 68 subjects will be needed in each group to reach a statistical power of 80%, assuming a SD of 2.5 in each group, with a significance level 0.05. A total sample size of 160 has been chosen to compensate for a probable 10% loss to follow-up.

### Participants and recruitment

A total of 160 participants will be recruited to the MODIAB-Web study performed in western and central Sweden. The enrollment started in two centers in November 2011 in the western region of Sweden. Successively, another four centers from the central part of Sweden were included; one was added in June 2012, another one in October 2012 and finally two more were included in June 2013. All study centers are hospital based. A list of participating study centers can be obtained upon request from the corresponding author.

One diabetes midwife in the antenatal care unit at each hospital-based study center identifies all pregnant women with type 1 diabetes. If women registered at the study units meet the inclusion criteria and do not meet any exclusion criteria, the midwife provides them with oral and written information about the study early in their pregnancy. If a woman agrees to participate in the study, she signs an informed consent form.

### Inclusion criteria

Participants have to fulfill the following criteria to be eligible for inclusion: literate and Swedish-speaking pregnant women over 18 years of age with a diagnosis of type 1 diabetes and registered at one of the six participating study centers, who have provided written informed consent beforehand.

### Exclusion criteria

Eligible participants will be excluded if they meet the following criterion: miscarriage before randomization, previously randomized to either the intervention or control group in the ongoing study, except randomized women who miscarry (first trimester) and are pregnant again during the data collection period; those who miscarried earlier but are now pregnant will be connected to the group to which they were randomized in the earlier pregnancy (intervention or control), and all data will be collected on this new pregnancy.

### Withdrawal criteria

Participants are withdrawn from the trial after randomization if they meet the following criteria: miscarriage, termination of pregnancy, intrauterine fetus death during the pregnancy or infant death within six months after birth.

### Ethical considerations

The study adheres to the principles of the Declaration of Helsinki [15] and has been approved by the Ethics Committee of Gothenburg, Sweden (No. 659-09). Written consent will be obtained from each participant after she has received written and verbal information about the study and before the baseline assessment. All participants have the right to withdraw from the study at any time.

### Randomization and allocation concealment

Participants are randomly allocated to the intervention group (IG) with web support with evidence-based information, self-care diary and communication with pregnant women with type 1 diabetes in addition to usual care, or to the control group (CG) provided only the usual care. Randomization is performed before baseline assessment.

The randomization is prepared with a unique code that consists of a first code letter identifying each center, a second code letter identifying which group it belongs to, and intervention (IG) or control (CG) followed by a consecutive number. Baseline questionnaires are marked with unique code letters/numbers and placed in a closed envelope. The envelopes are produced in blocks of ten, five IG and five CG. This is prepared by the research group and includes the single blind number. The allocation of participants will be equal (1:1) in blocks of ten for each of the six centers. After informed consent is received, the responsible diabetes midwife at each center gives the included woman a closed envelope and registers her personal information and the obtained code letters/numbers. The key connecting the code with the woman’s personal information is stored in a safe location at the study center, in accordance with that specific hospital’s policy. All data files used for data analysis contain the code number as the only source of identification.

### Blinding

Due to the nature of this trial it is not possible to blind the participants. Nevertheless, the diabetes midwives in charge of the random allocated sequence at the centers are not involved in the preparation of the envelopes, the use of web-based support or in the statistical analyses.

### Intervention

#### The web support group - intervention group

All of the women receive the usual care at one of the six study centers in Sweden. Each center has a special program for pregnant women with type 1 diabetes, and what constitutes usual care varies slightly between the care centers. However, all women have frequent contact with a midwife specialized in type 1 diabetes, an obstetrician and an endocrinologist during pregnancy and at one follow-up visit after childbirth.

In order to further support the women’s well-being and diabetes management, the intervention offers complementary web-based, person-centered support [16–18] in addition to usual care.

The web support springs from a holistic approach and consists of three components: 1) evidence-based information divided into three parts - being pregnant, labor and childbirth, and life as a new mother; 2) a self-care diary for self-reported monitoring of blood glucose, insulin doses, diet, activities and daily mood - measures can be viewed and evaluated in tables and diagrams to support decision making and self-care; and 3) a discussion forum for peer support divided into three parts - being pregnant, labor and childbirth, and life as a new mother - moderated by the research group. The website also provides a Frequently Asked Questions section as well as links to other sites that are relevant for childbearing women with type 1 diabetes.

The web-based information for pregnant women and new mothers with type 1 diabetes has been developed with a participatory design in seven different stages. The development process was driven by researchers, but it also engaged health-care professionals such as nurses, midwives, and physicians specializing in diabetes care, pediatrics, obstetrics and breastfeeding [18]. The web prototype containing the elements of the web support was further developed and tested by potential users, pregnant women and mothers with type 1 diabetes [16] before the web support program was launched.

The web-support system is used freely by the participants in the intervention group. There is no requirement regarding time spent logged into the system. If a participant in the intervention group has not accessed the web-based support within the last fortnight, a reminder is sent out via text message. The research group has no way of monitoring and/or limiting access to other web-based forums in relation to diabetes or childbearing during the study period. A minimum of two independent logons to the system is required for the participant to be considered to be adhering to the intervention.

#### Control group

The women in the CG receive the same usual care at one of the six study centers in Sweden (described above) as those in the IG.

### Follow-up period and outcome measurement

**Figure 2:**
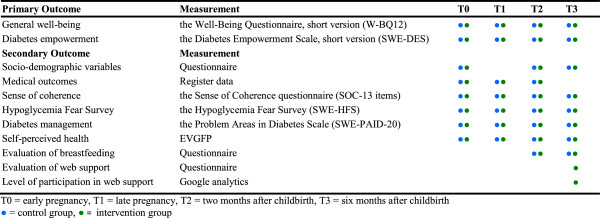
**Time periods for measurement of primary and secondary outcomes.**

During the 18-month follow-up period, the primary and secondary outcomes are tested at baseline, early pregnancy (T0), late pregnancy (T1), two months after childbirth (T2), and six months after childbirth (T3) (see Figure 2). Outcome measures are collected by questionnaires mailed to the participants in IG and CG. Participants who have not returned the questionnaire within ten days receive a phone call from the study coordinator or project assistant as a gentle reminder.

The study coordinator takes responsibility for the collection and entry of data into the correct computer files per protocol. Data collection will not be continued if a subject has been excluded from the study or has opted out. All data entry will be reviewed by two members of the research group who have not entered any of the data. The primary investigator (PI) has responsibility for the final data files and decides who will be granted access and for what purpose. The PI is also responsible for documenting and communicating any modifications in study protocol to all relevant parties. The sponsors and funders of this trial have no influence over any part of the study’s design or reporting of outcomes.

#### Primary outcome measures

The primary outcome of general well-being is evaluated with the Well-Being Questionnaire, short version (W-BQ12) [19] and diabetes empowerment with the Diabetes Empowerment Scale, short version (SWE-DES) [20].

#### Secondary outcomes measures

Coping in daily life is evaluated using the Sense of Coherence 13-item questionnaire (SOC-13 items) [21], and self-rated health will be assessed using a single-item EVGFP: excellent, very good, fair or poor. Diabetes management is measured with the Problem Areas in Diabetes Scale (SWE-PAID-20) [22]. Fear of hypoglycemia is assessed with the Hypoglycemia Fear Survey (SWE-HFS) [23]. The breastfeeding rate and experiences of nursing are evaluated with a specially developed questionnaire. Medical outcomes in terms of a glycated hemoglobin test (HbA1c) measured in early and late pregnancy and up to six months postpartum are collected from electronic medical records. Usefulness and usability of the web support are evaluated with a specially developed questionnaire and the level of participation by using Google Analytics. Socio-demographic variables, mode of delivery, diabetes duration and type of insulin administration are also recorded. All data collection forms listed above can be obtained from the corresponding author upon request.

### Validity and reliability of instruments used

The research group has opted to use well-tested instruments as outcome measures whenever possible. All instruments have to be available in Swedish to be used in this study. In the primary outcome measurements the W-BQ12 has a described Cronbach’s alpha of >0.88 [19] and its validity is considered to be good. The other primary outcome scale, SWE-DES, is considered acceptable in terms of reliability (Cronbach’s alpha range of 0.68 to 0.91 in the 23-question version of the scale) [20, 24]. In terms of secondary outcome, the Sense of Coherence 13-item scale is recognized as a reliable and valid instrument that can be applied across different cultures (range of Cronbach’s alpha 0.70 to 0.92 in 127 studies) [21]. The SWE-PAID-20 questionnaire was reported to have a (Cronbach’s alpha of 0.94) [25]. The SWE-HFS scale has also been reported as having good reliability (Cronbach’s alpha 0.85) and validity [22].

### Safety evaluation

The web-based support is not considered to cause any adverse event. The participants’ pregnancy statuses are monitored in order to avoid sending questionnaires to women who have experienced a lost pregnancy, and dates for delivery are registered to keep the deadlines for follow-up at two and six months post-partum.

### Trial duration

The inclusion period is estimated from November 2011 to December 2014 with follow-up until February 2016. See Table 1 for a time plan of the study and follow-up.

**Table 1:** **Time plan of the study and follow-up**

	Started	Will be completed in
Inclusion	November 2011	December 2014
Early pregnancy (T0)	November 2011	December 2014
Late pregnancy (T1)	April 2012	June 2015
Childbirth	June 2012	August 2015
2 months post partum (T2)	August 2012	October 2015
6 months post partum (T3)	November 2012	February 2016

### Statistical analyses plan

The full analysis set, Intention To Treat population (ITT population), consists of all randomized patients who had at least one follow-up measurement. The Per-Protocol population (PP population) consists of all patients in the ITT population without any significant protocol deviations. The PP population is defined at the clean-file meeting before the database is locked. Primary efficacy analyses will be performed on our two primary variables general well-being (W-BQ12) total score and diabetes empowerment (SWE-DES) total score, both at six months after delivery between the two randomized groups using Fisher’s non-parametric permutation test on the full analysis set (ITT population), two-sided test on significance level 0.025. If significant differences in baseline variables are found between the two randomized groups, a complementary analysis of covariance (ANCOVA) will be performed adjusted for these baseline variables. Last observation carry forward (LOCF) will be applied from two months if the six months value is missing.

Statistical analyses will be conducted using SPSS, version 16.0, and SAS, version 9.2 (Cary, NC). All tests will be two-tailed and conducted at 5% significance level.

## Discussion

The unique contribution of this project is the evaluation of the effectiveness of a specially designed web-based support for mothers with type 1 diabetes, which is expected to increase the women’s personal capacity, including knowledge, and thereby strengthen their autonomy during pregnancy, breastfeeding and early motherhood.

Web-based support systems have the potential to bridge gaps that can exist between the different health-care systems/professionals providing care during the childbearing period, and especially during early motherhood [13]. They can also offer increased convenience for users in terms of time, mobility, and geography, and promote equal access to quality-assured and evidence-based information [26]. Childbearing women in general are active Internet users [27, 28], and publications show that women with type 1 diabetes seek diabetes-related information online before, during, and after pregnancy [12, 13].

To our knowledge, there is a lack of studies evaluating web support with a more holistic approach to this group of women. According to a recent systematic review, there is limited evidence supporting the use of computer-based self-management interventions for adults with type 2 diabetes mellitus [29]. However, this group may not represent the most experienced Internet users compared to childbearing women with type 1 diabetes. The reasons for choosing the Internet as a venue for interventions have to be carefully considered regarding its contribution to improving people’s health [26]. From a methodological point of view, the review by Pal *et al*. [29] found interventions to be poorly understood and called for more detailed reporting and evaluation of the design, delivery and effectiveness of computer-based interventions. The study protocol describing the current RCT aims to contribute such detailed information about the study design and the measures undertaken to provide evidence for the use of web-based interventions in a patient group with specific needs during a vulnerable time of life.

### Strength and limitations

The strength of this study is that a generic web-based support is always accessible, can complement health care independently of differences in ‘usual care’ provided in each center, and places no extra burden on healthcare professionals. However, the use of the web-based support is voluntary, which might have an impact on the uptake of the intervention. We use the CONSORT-EHEALTH Checklist [29] to report on intervention adherence and measures undertaken to encourage the use of the web-based support; for example, an SMS reminder every second week. One limitation of the study is that it is not feasible to blind members of the healthcare team to group allocation, a common problem in clinical trials. Another limitation is that a higher percentage of women with type 1 diabetes suffer from miscarriage, which will prolong the data collocating period. Additional centers have been included to increase the data collection rate.

To conclude, we have described a protocol with a randomized controlled design in order to systematically evaluate the effectiveness of a web-based support to women with type 1 diabetes during pregnancy, childbirth and early motherhood. If proven effective, it will provide evidence for a modern and accessible complementary health-care intervention.

## Trial status

Status at time of submission of this article: recruiting.

## Abbreviations

ANCOVA: analysis of covariance
CG: control group
EVGFP: excellent, very good, fair or poor
HbA1c: glycated hemoglobin test
IG: intervention group
ITT population: intention to treat population
LOCF: last observation carry forward
PI: primary investigator
PP population: per-protocol population
RCT: randomized controlled trial
SOC-13 items: Sense of Coherence 13-item Scale
SWE-DES: Diabetes Empowerment Scale, short version
SWE-HFS: Hypoglycemia Fear Survey
SWE-PAID-20: Problem Areas in Diabetes Scale
W-BQ12: Well-Being Questionnaire, short version
pp: post-partum.

## References

[1] Azar M, Lyons T (2013). Management of pregnancy in women with type 1 diabetes. Minerva Endocrinol.

[2] Inkster ME, Fahey TP, Donnan PT, Leese GP, Mires GJ, Murphy DJ (2006). Poor glycated haemoglobin control and adverse pregnancy outcomes in type 1 and type 2 diabetes mellitus: systematic review of observational studies. BMC Pregnancy Childbirth.

[3] Kinsley B (2007). Achieving better outcomes in pregnancies complicated by type 1 and type 2 diabetes mellitus. Clin Ther.

[4] Hawdon JM (2011). Babies born after diabetes in pregnancy: what are the short- and long-term risks and how can we minimise them?. Best Pract Res Clin Obstet Gynaecol.

[5] Soltani H, Dickinson FM, Kalk J, Payne K (2008). Breast feeding practices and views among diabetic women: a retrospective cohort study. Midwifery.

[6] Berg M (2005). Pregnancy and diabetes: How women handle the challenges. J Perinat Educ.

[7] Berg M, Honkasalo M (2000). Pregnancy and diabetes–a hermeneutic phenomenological study of women’s experiences. J Psychosom Obstet Gynaecol.

[8] Berg M, Sparud-Lundin C (2009). Experiences of professional support during pregnancy and childbirth - a qualitative study of women with type 1 diabetes. BMC Pregnancy Childbirth.

[9] Sparud-Lundin C, Berg M (2011). Extraordinary exposed in early motherhood - a qualitative study exploring experiences of mothers with type 1 diabetes. BMC Womens Health.

[10] Berg M, Erlandsson L-K, Sparud-Lundin C (2012). Breastfeeding and its impact on daily life in women with type 1 diabetes- a prospective cohort study. Int Breastfeed J.

[11] Berg M, Sparud-Lundin C (2012). Well-being, diabetes management, and breastfeeding in women with type 1 diabetes two and six month after childbirth. J Womens Health Care.

[12] Rasmussen B, Hendrieckx C, Clarke B, Botti M, Dunning T, Jenkins A, Speight J (2013). Psychosocial issues of women with type 1 diabetes transitioning to motherhood: a structured literature review. BMC Pregnancy Childbirth.

[13] Sparud-Lundin C, Ranerup A, Berg M (2011). Internet use, needs and expectations of web-based information and communication in childbearing women with type 1 diabetes. BMC Med Inform Decis Mak.

[14] Zwarenstein M (2008). Improving the reporting of pragmatic trials: an extension of the CONSORT statement. BMJ.

[15] World Medical Association (2013). World medical association declaration of Helsinki ethical principles for medical research involving human subjects. JAMA.

[16] Adolfsson A, Jansson M (2012). Prototype for Internet support of pregnant women and mothers with type 1 diabetes: focus group testing. Psychol Res Behav Manag.

[17] Berg M, Adolfsson A, Ranerup A, Sparud-Lundin C (2013). Person-centered Web support to women with type 1 diabetes in pregnancy and early motherhood–the development process. Diabetes Technol Ther.

[18] Linden K, Berg M, Sparud-Lundin C (2012). Web-based information for pregnant women and new mothers with type 1 diabetes - a descriptive of the development process. BMC Med Inform Decis Mak.

[19] Pouwer F, van der Ploeg HM, Adèr HJ, Heine RJ, Snoek FJ (1999). The 12-item well-being questionnaire: an evaluation of its validity and reliability in Dutch people with diabetes. Diabetes Care.

[20] Wikblad K, Andersson B (2005). Swe-DES-23 - Swe-DES-SF-10 Den svenska versionen av Diabetes Empowerment Scale (Swe-DES).

[21] Eriksson M, Lindström B (2005). Validity of Antonovsky’s sense of coherence scale: a systematic review. J Epidemiol Community Health.

[22] Anderbroa T, Amsberga S, Wredlinga R, Linsa P-E, Adamsona U, Lisspersc J, Johanssona U-B (2008). Psychometric evaluation of the Swedish version of the Hypoglycaemia fear survey, short communication. Patient Educ Couns.

[23] Gonder-Frederick LA, Schmidt KM, Vajda KA, Greear ML, Singh H, Shepard JA, Cox DJ (2011). Psychometric properties of the hypoglycemia fear survey-II for adults with type 1 diabetes. Diabetes Care.

[24] Leksell J, Funnell M, Sandberg G, Smide B, Wiklund G, Wikblad K (2007). Psychometric properties of the Swedish diabetes empowerment scale. Scand J Caring Sci.

[25] Amsberg S, Wredling R, Lins PE, Adamson U, Johansson UB (2008). The psychometric properties of the Swedish version of the problem areas in diabetes scale (Swe-PAID-20): scale development. Int J Nurs Stud.

[26] Griffiths F, Lindenmeyer A, Powell J, Lowe P, Thorogood M (2006). Why are health care interventions delivered over the internet? A systematic review of the published literature. J Med Internet Res.

[27] Lagan B, Sinclair M, Kernohan W (2010). Internet use in pregnancy informs women’s decision making: a web-based survey. Birth.

[28] Larsson M (2009). A descriptive study of the use of the Internet by women seeking pregnancy-related information. Midwifery.

[29] Pal K, Eastwood SV, Michie S, Farmer AJ, Barnard ML, Peacock R, Wood B, Inniss JD, Murray E (2013). Computer-based diabetes self-management interventions for adults with type 2 diabetes mellitus (Review). Cochrane Database Syst Rev.

